# Sources of Information on Medicinal Products Among Physicians – A Survey Conducted Among Primary Care Physicians in Poland

**DOI:** 10.3389/fphar.2021.801845

**Published:** 2022-01-06

**Authors:** Magdalena Zielińska, Tomasz Hermanowski

**Affiliations:** Department of Bioanalysis and Drug Analysis, Faculty of Pharmacy, Medical University of Warsaw, Warsaw, Poland

**Keywords:** primary care physicians, drug information sources, information-seeking behaviours, barriers, prescribing attitudes

## Abstract

**Introduction:** Primary care physicians need to have access to up-to-date knowledge in various fields of medicine and high-quality information sources, but little is known about the use and credibility of sources of information on medicinal products among Polish doctors. The main goal of this study was to analyze the sources of information on medicinal products among primary care physicians in Poland.

**Methods:** A survey was conducted among 316 primary care physicians in Poland. The following information was collected: demographic data of participants, type and frequency of using data sources on medicinal products, barriers to access credible information, assessment of the credibility of the sources used, impact of a given source and other factors on prescription decisions.

**Results:** The most frequently mentioned sources of information were medical representatives (79%), medical journals (78%) and congresses, conventions, conferences, and training (76%). The greatest difficulty in finding the latest information about medicinal products was the lack of time. The surveyed doctors considered clinical guidelines to be the most credible source of information, and this source also had the greatest impact on the choice of prescribed medicinal products.

**Conclusion:** The study showed that clinicians consider clinical guidelines as the most credible source of information with the greatest impact on prescribing medicinal products. However, it is not the source most often mentioned by doctors for obtaining knowledge about medicinal products. There is a need to develop strategies and tools to provide physicians with credible sources of information.

## Introduction

Evidence-based medicine (EBM) aims to improve patient outcomes and provides a solid scientific basis for making clinical decisions (Sackett et al., 1996; Timmermans and Mauck, 2005). According to EBM, patient care should be based on the best available scientific evidence and combine clinical knowledge with the needs and preferences of patients. The goal of EBM is safer, more consistent, and more cost-effective care (Greenhalgh et al., 2014), which translates into a reduction in health care costs and cognitive overload that can lead to medical errors as well as an increase in the effectiveness and quality of health services (Gillam and Siriwardena, 2014). The amount of new scientific knowledge about effective, efficient and safe patient care is constantly growing, while the established knowledge is becoming outdated (Grol, 2001). Following up with new evidence and implementing it in the daily care of patients ensures high-quality healthcare, but it constitutes a significant challenge for all areas of medicine. It is especially important for primary care physicians who require a large amount of up-to-date knowledge in all areas of medicine (Nylenna and Aasland, 2000).

As spending on pharmaceutical products is typically the largest cost component in outpatient care (Godman et al., 2009; Godman et al., 2010a; Godman et al., 2010b; Sermet et al., 2010; Vončina et al., 2011; Godman et al., 2012a), and these costs increase faster than other cost types in this sector (Godman et al., 2009; Godman et al., 2010a; Godman et al., 2012b), providing reliable, credible and best-available drug information can help in optimizing incurred expenses. Scientific reports show that recommendations are often not applied in practice (Grol, 2001; Schuster et al., 2005) resulting in gaps between EBM and clinical reality (Carlsen and Norheim, 2008). As a result, we may encounter misuse of medicinal products, which increases the risk of therapeutic failure, adverse events, antimicrobial resistance, and a waste of resources (Bergman and Wiholm, 1981; Mölstad et al., 2008; Norman et al., 2009; McGinn et al., 2010). Prescribing medicinal products is a complicated decision-making process, taken both by the physician and partially by the patient (Jackson et al., 2004; Thistlethwaite et al., 2010). To be successful in implementing best practices, a systematic approach and proper planning are essential. With a better understanding of the prescribing process and the factors influencing it, it is easier to design ways to implement the best current scientific evidence in clinical practice.

In Poland, the rationalization of pharmacotherapy is emphasized by the Minister of Health in the National Health Policy. As the variety of pharmacotherapeutic choices grow rapidly, the percentage of drug expenditure in total medical costs is increasing as well, exceeding the capacity of patients and public budgets. In the face of these changes, promoting EBM is essential. Prescription decisions are supported by local and international scientific societies, which develop recommendations elaborated by experts in a given field. Yet, the use of guidelines in Poland is not obligatory nor supported by financial incentives. Nevertheless, there are ongoing efforts to promote the evidence-based clinical practice and to systematize the process of guideline development. New solutions have been introduced to standardize the development and updating of the guidelines in line with internationally recognized methodology. This process involves public institutions such as the Agency for Health Technology Assessment and Tariff System (Polish: Agencja Oceny Technologii Medycznych i Taryfikacji, AOTMiT). Initiatives to support the development and use of the guidelines so far included the development of diagnostic and therapeutic pathway recommendations, training and promotion of evidence-based decisions among health care professionals, standardization of therapeutic interventions, and eliminating therapeutic approaches with unproven efficacy. However, there is still a lack of actions supporting the use of credible sources of drug information (SoI) in daily medical practice. As this gap exists, our study aimed to support activities promoting the use of EBM for prescription decisions by investigating how physicians are using available SoI on medicines and by identifying the most common barriers of this process. This may help showing the problem to a greater audience as well as sharing the local experience with other countries that struggle to improve the rational use of medications. Information sources used by physicians have been described as one of the main determinants of the quality of the prescribing process (Figueiras et al., 2000). Research shows that one of the obstacles to compliance by physicians is the lack of knowledge of these guidelines (Cabana et al., 1999), therefore it seems important to promote those SoI which are based on EBM. This involves a prior analysis of the actual preferences and behaviour of the target group, on the basis of which the strategy for both dissemination and implementation can be selected.

The main goal of this study was to analyze the SoI on medicinal products among primary care physicians in Poland. The study aimed to give insight into the following topics: frequency and type of SoI used, determination of the credibility with which physicians perceive the SoI used, determination of factors and type of SoI with the greatest impact on the selection of medicinal products prescribed to patients, as well as identification of barriers hindering access to the latest knowledge about medicinal products.

## Materials and Methods

### Participants

An anonymous, voluntary survey of primary care physicians was conducted from May to June 2019. After giving informed consent to participate in the study, the respondents received a questionnaire from the interviewer with detailed instructions on how to complete it, and they were assured that all individual responses would be treated as confidential. We aimed to recruit 5% of active primary care physicians in the target area. As a result, to this study, 316 primary health care physicians were enrolled from the Mazowieckie voivodship because the survey was carried out by the interviewers personally. The questionnaire is presented in Supplementary Table S1. The study protocol was approved by the Commission of Bioethics at Medical University of Warsaw (AKBE/238/2019). The study did not contain any identifiable human or animal data.

### Questionnaire

The survey consisted of nine single-choice and multiple-choice questions, as well as questions in which the pool of 100 points had to be divided between the given answers. The first part of the survey included questions about demographic data—gender and age of the respondents. The next questions concerned the self-assessment of the respondents in relation to their knowledge about medicinal products as well as the type and frequency of using data SoI on medicinal products in the last 6 months. In the last part of the questionnaire, information was collected on the causes that hinder access to credible information about medicinal products, the doctors’ assessment of the credibility of the SoI used and the influence of a given SoI on the choice of the product prescribed to the patient, as well as the influence of other factors on prescription decisions.

The questionnaire was based on the one developed in 2017 to enable changes over time, but before sharing it with the study sample, the questionnaire was consulted and tested. First, the questionnaire was verified for reliability and accuracy by health care experts and a market research expert. As a result, the drug information sources were updated according to the current availability and the questionnaire was shortened from 10 to 9 questions. Next, the pilot phase was performed with 10 primary health care physicians practising in the same area. The aim of the pilot phase was to evaluate whether the questionnaire was legible, relevant, answers comprehensive, and of acceptable length. The physicians confirmed that the questionnaire was legible, relevant, the questions and response options were clearly formulated, understandable and comprehensive, the length of the questionnaire was appropriate and it was not difficult to complete. The questionnaire was then repeated on the same study group after 2 weeks for further validation. The data obtained were subjected to statistical analysis. The Wilcoxon test was conducted to verify differences between responses to individual questions in the first and second measurements. The Wilcoxon test showed that the responses of the surveyed doctors in both measurements were statistically similar to each other. There were only three exceptions concerning the responses: “handy indexes of drugs” in question 7, “foreign databases on the Internet” in question 8 and “data related to the use of the drug” in question 9. It is important to emphasise a significant limitation of this analysis, which is the sample size. Therefore, a qualitative evaluation of the questionnaire was taken as the main objective of the pilot phase. After pilot phase, the questionnaire was accepted for use in this study. Physicians participating in the pilot phase were not included in the analysis.

### Statistical Analysis

Data were statistically analysed. The chi-square test was used for categorical data to compare the percentage of respondents indicating specific answers by gender or age category.

For questions, in which the respondents were asked to assign a certain non-negative number of points to every possible answer, so the sum of points for all the answers totalled up to 100, the Kruskal – Wallis test was used.

In the case of questions 7 and 8 (Supplementary Table S1), the number of respondents indicating a given SoI as the highest-scored were counted. If the respondent rated k > 1 SoI at the same maximum level, then all were counted as maximum with a weight of 1/k.

The Pearson correlation coefficient with a 95% confidence interval was used to assess correlations between the answers to the following questions:

• Assessment of the credibility of individual SoI (question 7),
• Impact of these SoI on decisions about prescribing medicinal products to patients (question 8).

The correlation was estimated at the general level: the average score of “relative credibility” vs the average score of “impact on decisions on prescribing drugs” across all SoI.

Differences and correlations were considered statistically significant if *p* values were below 0.05.

In the case of questions 6–9, in which the respondents of each possible answer assigned a certain number of non-negative points, so the sum of points for all answers added up to 100, it was necessary to make some adjustments to the input data:

• In question 6, simultaneous non-zero scores were considered mutually exclusive if given the answer “no difficulties” and to some other answer (indicating some difficulties). In such cases, if the respondent noticed any difficulties, the score for no difficulties was changed to zero,
• In questions 6–9 when the sum of points for individual answers was different from 100), the points for all answers were proportionally scaled so that they sum up to 100.

The analysis was performed using R version 3.6.3 (R Foundation for Statistical Computing, Vienna, Austria) and MS Excel 2016 (Microsoft Corp., Redmond, WA, United States).

## Results

Overall, 72% of women and 28% of men took part in the study. The respondents represented various age groups. Among the respondents, there were 18% of people aged 25–35 years, 19% aged 36–45 years, 31% aged 46–55 years, 21% aged 56–65 years, and 11% of the respondents belonged to the group of people over 65 years of age.

In total, 22% of the respondents (24% of women and 18% of men) declared a feeling of lack of information about medicinal products in their practice. Among the 10 categories of SoI on medicinal products (Table 1), the surveyed doctors indicated, on average, six of them. The most frequently mentioned SoI were medical representatives (79% of respondents), medical journals (78% of respondents) and congresses, conventions, conferences and trainings (76% of respondents), the least frequently foreign on-line databases and medical portals (18% of respondents) and information from the Ministry of Health and AOTMiT (18% of respondents) (Table 1).

**TABLE 1:** Sources of information on medicinal products used by responding physicians in the last 6 months (*N* = 316).

Data sources on medicinal products	Respondents who named the source*n* (%)
Medical representatives	250 (79)
Medical journals	248 (78)
Congresses, conventions, stationary conferences, interactive conferences/on-line training	239 (76)
National medical portals (e.g., Medycyna Praktyczna, MEDtube), mobile applications (e.g., Bartosz Talks, eMPedium), online forums for doctors (e.g., Konsylium24)	222 (70)
Clinical guidelines of medical associations	205 (65)
Handy drug indexes (e.g., “Reimbursed Medicines List,” “Pharmindex”)	165 (52)
Medical books	149 (47)
Knowledge and experience of colleagues from work	148 (47)
Foreign databases on the Internet (e.g., “Medline”), medical portals	56 (18)
Information from the Ministry of Health, reports of the Agency for Health Technology Assessment and Tariffs	56 (18)

The question about the frequency of using SoI on medicinal products over the last 6 months showed that nearly half (49%) of the surveyed physicians used SoI on medicinal products several times a day, 39% several times a week, 11% several times a month and only 1% of respondents declared that they use SoI on medicinal products several times every 6 months (Table 2).

**TABLE 2:** The declared frequency of using sources of information on medicinal products in the last 6 months (*N* = 316).

Frequency of using sources of information on medicinal products during the last 6 months	Number of respondents*n* (%)
Several times a day	155 (49)
A few times a week	122 (39)
Several times a month	36 (11)
Several times in 6 months	3 (1)
I have not used	0

The relationship between the frequency of using SoI about medicinal products and the age of the respondents was analyzed. It has been shown that significantly more people in the 25–45 age group (62% of people from the given age group) used SoI about medicinal products several times a day compared to people aged 46+ (42% of people in this age group), *p* < 0.01.

**FIGURE 1:**
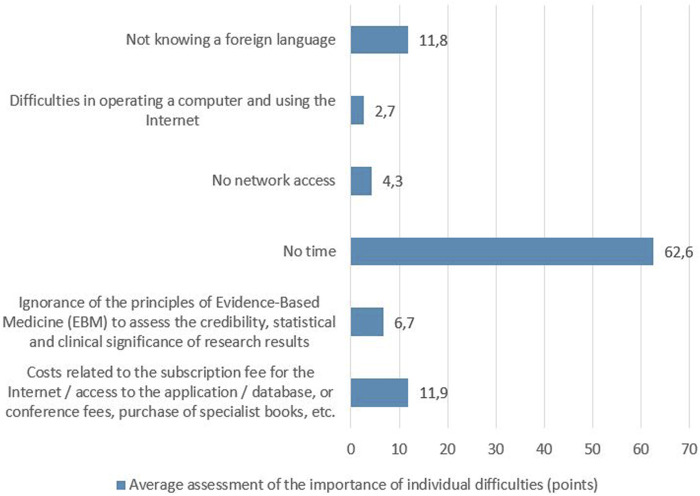
Average assessment of the importance of individual difficulties.

When asked about the causes that make it difficult to obtain the latest information about medicinal products, 110 respondents (34%) declared that they did not notice any difficulties. Among the remaining respondents (*n* = 206) who reported difficulties, the greatest was the lack of time (62.6 points on average), followed by costs (access to applications, databases, books, conference fees) and not knowing a foreign language (11.9 and 11.8 points, respectively) – Figure 1. Analysis of limitations between age categories showed that the respondents aged 65+ were significantly more likely to report difficulties in operating a computer and using the Internet than younger people. In contrast, they were significantly less likely to report costs related to the subscription fee for the Internet/access to the application/database, or conference fees, purchase of specialist books, etc as a barrier. These differences were statistically significant, *p* < 0.01.

**FIGURE 2:**
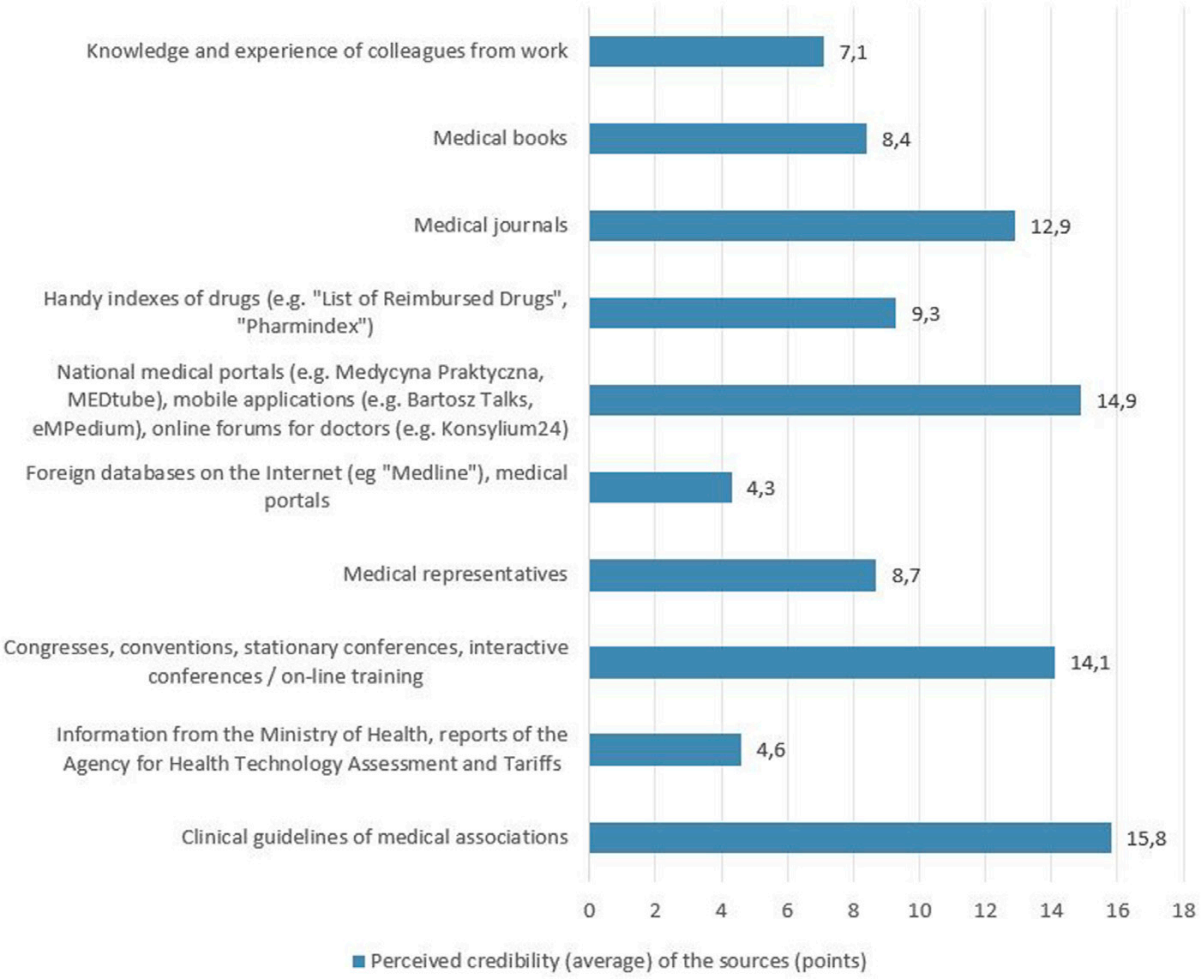
Perceived credibility (average) of the sources of information on medicinal products as assessed by the surveyed doctors.

**FIGURE 3:**
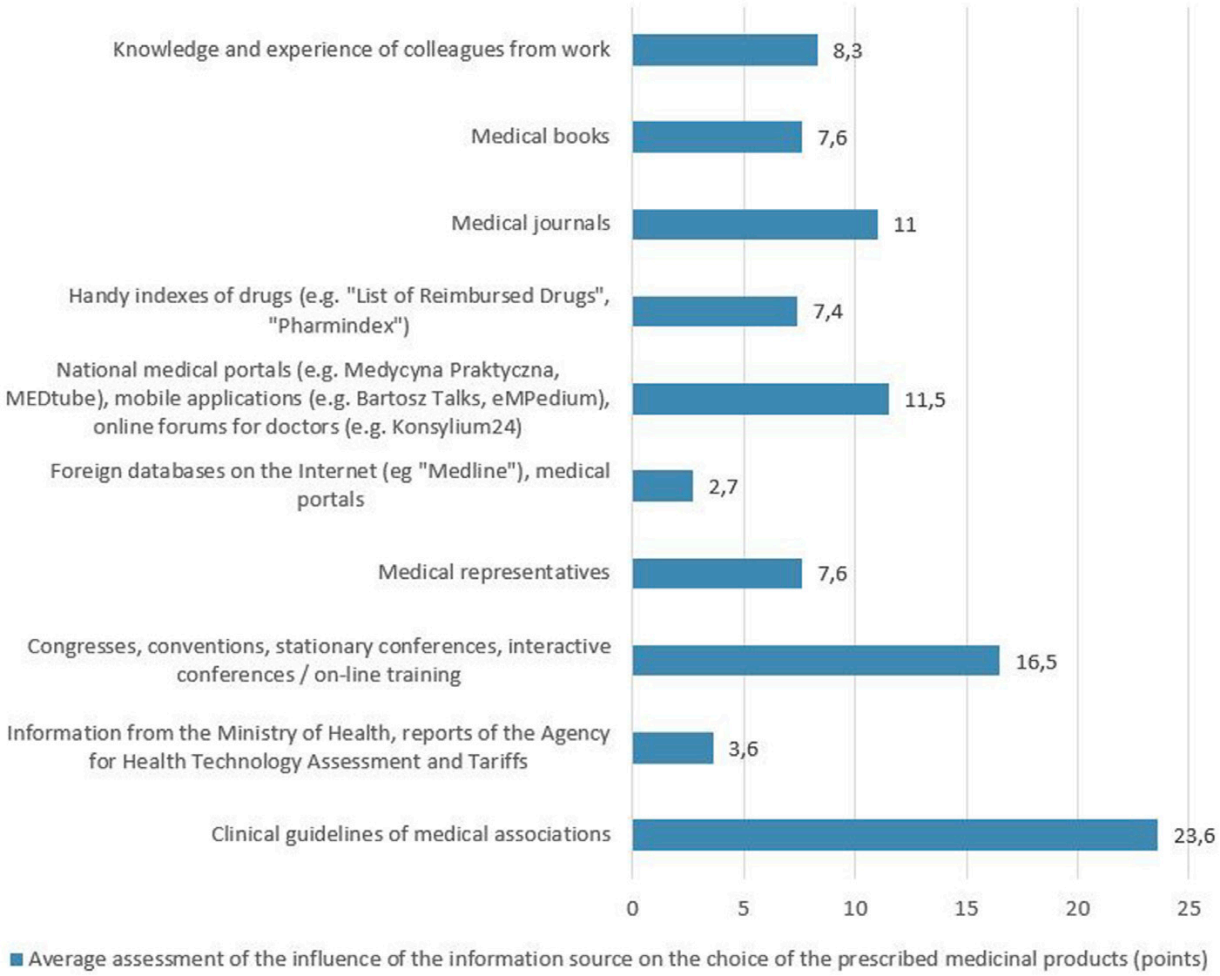
Average assessment of the influence of the information source on the choice of the prescribed medicinal products.

The respondents were also asked which of the 10 mentioned SoI about medicinal products they consider the most credible. The responses showed that clinical guidelines (15.8 points on average), national medical portals (14.9 points on average) and congresses, conventions, conferences and training (14.1 points on average) were considered the most credible, while the least credible was the information provided by the Ministry of Health, AOTMiT reports (4.6 points on average) and foreign on-line databases and medical portals (4.3 points on average) - Figure 2. As part of the described study, it was analyzed which of the given SoI about medicinal products has the greatest impact on the choice of the medicinal product prescribed for the patient. The SoI which, based on the respondents’ answers, had the greatest impact on the choice of the prescribed medicinal product were clinical guidelines (23.6 points on average) and congresses, conventions, conferences and training (16.5 points on average), while the least influence was attributed to the information of the Ministry of Health, AOTMiT reports (3.6 points on average) and foreign on-line databases and medical portals (2.7 points on average) - Figure 3.

The respondents were also asked to evaluate which factors—data related to the use of the medicinal product (indications, side effects, interactions, etc.), guidelines that are the source of the best currently known procedures or costs of pharmacotherapy—have the greatest influence on the choice of the medicinal product prescribed for the patient. The average assessment of the influence of individual factors on the selection of the prescribed medicinal product was 47.1 points for data related to the use of the medicinal product and 41.5 points for the guidelines, while the cost of pharmacotherapy was considered by the respondents to be the factor with the lowest impact on prescription decisions (16.8 points).

**FIGURE 4:**
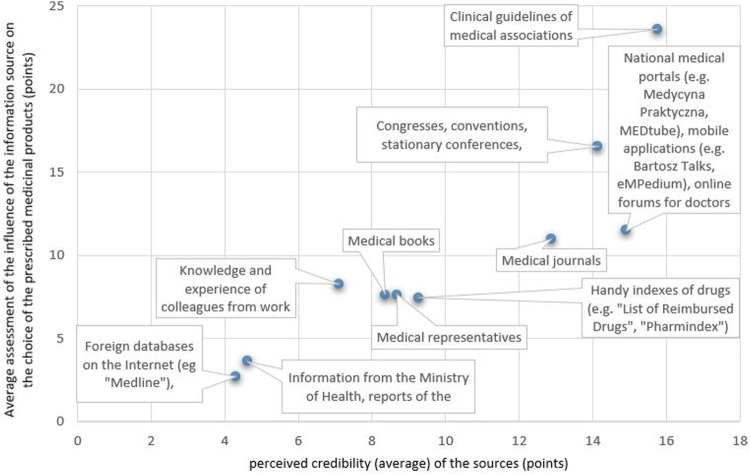
Correlation between the perceived credibility of a source of information about a medicinal product and the influence of the source on the choice of a prescribed medicinal product.

The analysis of the correlation between the perceived credibility of the SoI about the medicinal products and the influence of the SoI on the choice of the prescribed medicinal product (Figure 4) showed that the SoI perceived by the respondents to be more credible have a greater impact on the selection of the prescribed medicinal product, the Pearson correlation coefficient *r* = 0.88, 95% CI (0.56; 0.97).

**FIGURE 5:**
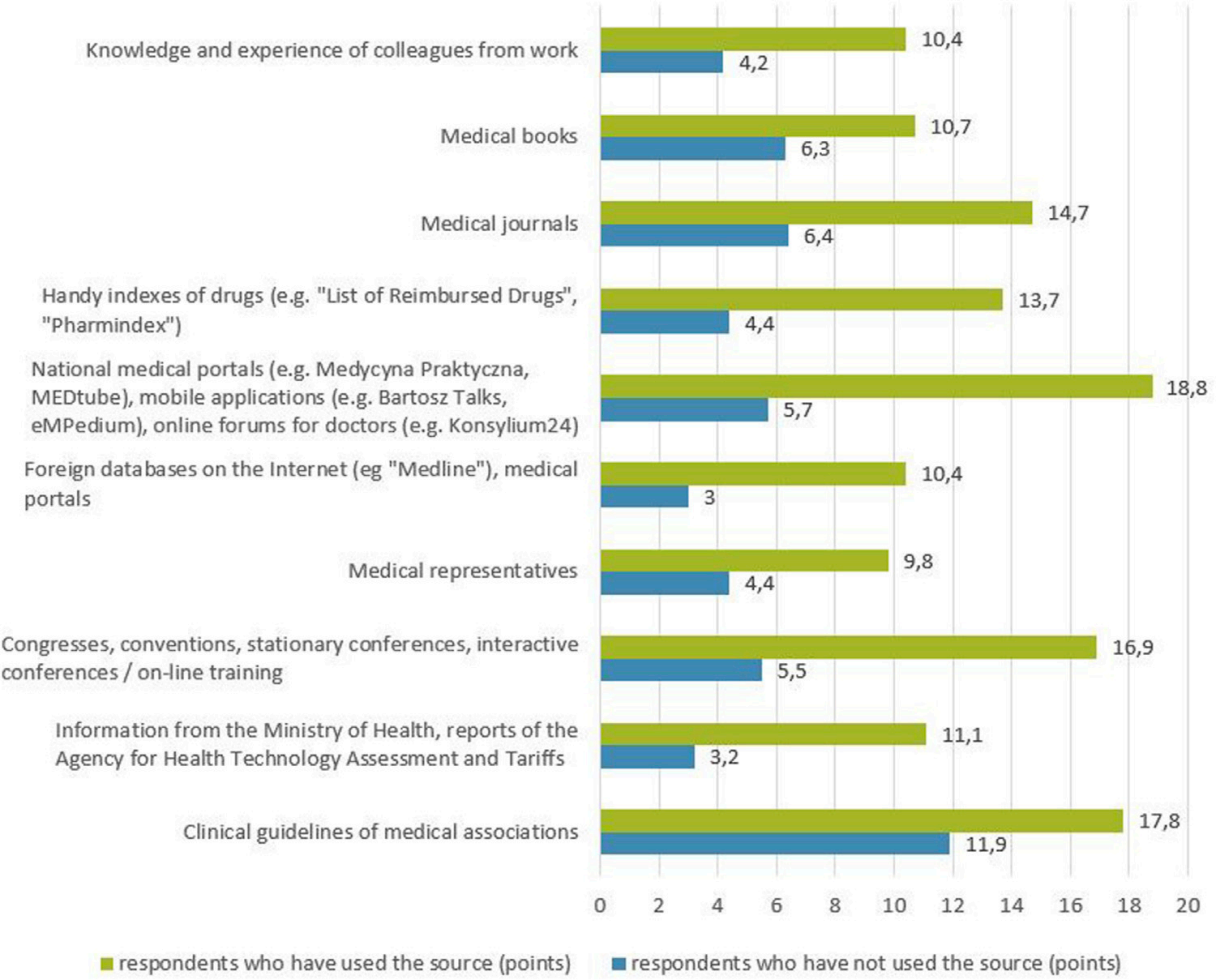
Relationship between the declaration of using a source of information about a medicinal product in the last 6 months and its perceived credibility (points).

The analysis of the relationship between the declaration of using a SoI about the medicinal product in the last 6 months and its perceived credibility, showed that this relationship does exist (Figure 5). Namely, the average rating of the credibility of a given SoI in the opinion of respondents is higher in the group of respondents declaring the use of this SoI than among respondents who have not used the source for the last 6 months (for each of the sources of information *p* < 0.01).

## Discussion

This study analyzed the use of SoI about medicinal products among primary care physicians in Poland. We found that only 22% of respondents noticed the lack of information about medicinal products in their practice. The surveyed physicians most often mentioned medical representatives, medical journals as well as congresses, conventions, conferences and trainings as SoI on medicinal products used in the last 6 months.

Studies on how primary care physicians access information about medicinal products were conducted in other countries as well providing similar results. An analysis of the practices of obtaining information showed that Norwegian physicians prefer congresses and courses (Nylenna and Aasland, 2000), while Canadian doctors opt for medical journals as the most frequently used SoI (Kosteniuk et al., 2013). The exceptions are medical representatives who, in similar studies, are cited as a less frequently used SoI (González-González et al., 2007; Kosteniuk et al., 2013) and are perceived as less important (Nylenna and Aasland, 2000; Le, 2017). This difference may result from poorly developed methods of providing doctors with information from independent sources in Poland. There is a lack of appropriate tools and an approach based on the promotion and dissemination of clinical practice guidelines or other educational materials that would be developed in an accessible form by independent organizations. The SoI about medicinal products of less importance were national medical portals and clinical guidelines (70 and 65% of respondents, respectively). The rating of SoI is consistent with the reports from Canada, which also point to clinical practice guidelines and websites and Danemark, which mention the role of medical representatives (Le, 2017). The SoI about medicinal products that were much less frequently mentioned in our study were medical books and the knowledge and experience of colleagues from work. These data differ from the available studies which showed that colleagues and textbooks are one of the most commonly used SoI (González-González et al., 2007; Jones et al., 2007; Fadare et al., 2019) which may be due to the size of the practice and the ability to freely consult information on medicinal products with other doctors. Information from the Ministry of Health and AOTMiT reports were some of the least frequently mentioned SoI by the respondents. Moreover, these sources were perceived as the least credible, having little influence on the choice of the prescribed medicinal product, which is a very disturbing result. The obtained data show that it is necessary to take measures to improve the confidence of doctors in the information presented on the website of state institutions and to improve the effectiveness of disseminating information prepared by these institutions.

An analysis of the frequency of using SoI about medicinal products showed that the majority of respondents (88%) use them at least several times a week, which may indicate a high need to update knowledge and confirm their decisions in available sources. It is also an area with significant potential for positive changes to improve the quality of care provided because providing doctors with reliable, credible and easy-to-use SoI that will be often used by doctors can positively affect the quality of prescriptions and services provided and reduce expenditure on healthcare.

The analysis of difficulties in finding the latest information about medicinal products showed that one-third of the respondents declared that they did not notice any difficulties accessing information about medicinal products, while the others indicated the lack of time, costs, and not knowing a foreign language as barriers to access credible information about medicinal products. The lack of time—the greatest barrier found in our study was also identified in other studies (McColl et al., 1998; Andrews et al., 2005; Cook et al., 2013).

When analysing the credibility of the SoI on medicinal products, clinical guidelines, and then national medical portals as well as congresses, conventions, conferences and training courses, are considered the most credible SoI in the opinion of the respondents. Although clinical guidelines were indicated as the most credible SoI, they were not the most frequently used. This may be rooted in the volume of clinical guidelines, which makes them difficult and time-consuming while using.

Factors with the greatest impact on the selection of the product prescribed for the patient were also investigated. The choice of the prescribed medicinal product was most influenced by clinical guidelines of medical associations and characteristics of the medicinal product (indications, side effects, interactions, etc.). Different results in this regard were obtained in a study of Canadian doctors, which showed that medical textbooks and then colleagues are the most popular SoI influencing clinical decisions (Kosteniuk et al., 2013), as well as a study of Italian primary care physicians, in which medical textbooks and journals, as well as fellow physicians, are the most frequently used SoI when making prescription decisions (Maio et al., 2011). An important role of clinical guidelines suggested by our study along with their perceived credibility gives helpful guidance for the development of educational materials for physicians, which would be based on clinical guidelines and present the most important issues briefly or schematically. This solution would enable physicians to use SoI about medicinal products which, in their opinion, are credible and have the greatest impact on the choice of the prescribed medicinal product, but at the same time do not require a lot of time commitment.

The positive correlation between the perceived credibility of the SoI about a medicinal product and the influence of the SoI on the choice of the prescribed medicinal product indicates the greater importance of SoI perceived as more credible in the prescribing decisions. Our study also showed a positive correlation between the declared use of a SoI about the medicinal product in the last 6 months and its perceived credibility. According to the results, the average assessment of the perceived credibility of the SoI was higher in the group of physicians declaring the use of this SoI than among the respondents who did not use a given SoI for the last 6 months.

Our study may be limited by the location where the surveyed doctors provided their services. Most of the respondents worked in Warsaw, which is the capital of Poland, where doctors have wide access to academic centres, conferences, meetings and speeches of specialists. The preferences and difficulties among doctors working in smaller cities and other provinces, with worse access to SoI about medicinal products, may differ from those obtained in this study. A second limitation to consider is that survey results may be biased—respondents may only want to say what will be well perceived and what the interviewer or the public expects to hear.

The literature indicates that general practitioners prefer SoI that are readily available, applicable to general practice, easy to use, and of high quality (Dawes and Sampson, 2003). Further analysis of this area is important as it may enable better-targeted methods to promote credible SoI about medicinal products in clinical practice and consequently improve the quality of healthcare. Research to date has shown that there are no “magic bullets” to improve quality in healthcare (Oxman et al., 1995), but many interventions are available, such as academic detailing (Costa et al., 2016), printed educational materials (Giguère et al., 2020), computerized (Arditi et al., 2017) or manually (Pantoja et al., 2019) generated reminders delivered in a printed form, financial incentives (Wettermark et al., 2009), interventions that increase the use of cheaper generic medicinal products (Vončina et al., 2011; Godman et al., 2014; Martin et al., 2014; Leporowski et al., 2018) and drug recommendations in the form of a “Wise List” (Gustafsson et al., 2011), which, if properly applied, can constitute an effective intervention for potentially important influence on the improvement of the professional practice of doctors and contribute to significant savings in health care expenses. According to the available literature, the decisions of family doctors to change prescription habits are associated with many factors (Armstrong et al., 1996). The information obtained in this study may be helpful in the future development and dissemination of credible SoI about medicinal products among primary care physicians in Poland.

## Data Availability

The raw data supporting the conclusion of this article will be made available by the authors, without undue reservation.
