# Supplementary material for: Sources of Information on Medicinal Products Among Physicians – A Survey Conducted Among Primary Care Physicians in Poland
Source: Front Pharmacol. 2022 Jan 6;12:801845. doi: 10.3389/fphar.2021.801845 (PMC8770910; doi:10.3389/fphar.2021.801845)
Supplement: Supplementary file 1 [file Table1.DOCX]

Supplementary Material

Supplementary Table 1.

| 1. **Please select as appropriate:**  - Women - Men  1. **What is your age range:**  - 25 – 35 Years of Age - 36 – 45 Years of Age - 46 – 55 Years of Age - 56 – 65 Years of Age - Over 65 Years of Age  1. **Do you feel there is a lack of information about drugs in your practice:**  - Yes - No  1. **Please indicate the sources of information on medicines used by you in the last six months (several answers possible):**  - Knowledge and experience of work colleagues - Medical books - Medical journals - Handy indexes of drugs (eg "List of Reimbursed Drugs", "Pharmindex") - National medical portals (e.g. Medycyna Praktyczna, MEDtube), mobile applications (e.g. Bartosz Talks, eMPedium), online forums for doctors (e.g. Konsylium24) - Foreign databases on the Internet (eg "Medline"), medical portals - Medical representatives - Congresses, conventions, stationary conferences, interactive conferences / on-line training - Information from the Ministry of Health, reports of the Agency for Health Technology Assessment and Tariffs - Clinical guidelines of medical associations |
| --- |
| 1. **How often have you used sources of information on medicines in the last six months (mark only one answer):**  - Several times a day - Several times a week - Several times a month - Several times every six months - I have not used any  1. **In your practice, are there any reasons that make it difficult to obtain the latest information about drugs? If there are difficulties, which of the factors listed above limits your access to current medical reports to the greatest extent (please divide the pool of 100 points between the following answers, assuming that 100 is the greatest and 0 is the smallest restriction):**  - Not knowing a foreign language - Difficulties in operating a computer and using the Internet - No network access - No time - Ignorance of the principles of Evidence-Based Medicine (EBM) to assess the credibility, statistical and clinical significance of research results - Costs related to the subscription fee for the Internet / access to the application / database, or conference fees, purchase of specialist books, etc. - No difficulties  1. **Which sources of information are the most credible for you (please divide the pool of 100 points between the following answers, assuming that 100 is the most credible source of data and 0 the least credible):**  - Knowledge and experience of colleagues from work - Medical books - Medical journals - Handy indexes of drugs (e.g. "List of Reimbursed Drugs", "Pharmindex") - National medical portals (e.g. Medycyna Praktyczna, MEDtube), mobile applications (e.g. Bartosz Talks, eMPedium), online forums for doctors (e.g. Konsylium24) - Foreign databases on the Internet (eg "Medline"), medical portals - ̤Medical representatives - ̤Congresses, conventions, stationary conferences, interactive conferences / on-line training - Information from the Ministry of Health, reports of the Agency for Health Technology Assessment and Tariffs - Clinical guidelines of medical associations  1. **Which source of information has the greatest impact on your decision about the type of drug prescribed to the patient (please divide the pool of 100 points between the following answers, assuming that 100 has the greatest and 0 the least influence on the prescribed preparation):**  - Knowledge and experience of colleagues from work - Medical books - Medical journals - Handy indexes of drugs (e.g. "List of Reimbursed Drugs", "Pharmindex") - National medical portals (e.g. Medycyna Praktyczna, MEDtube), mobile applications (e.g. Bartosz Talks, eMPedium), online forums for doctors (e.g. Konsylium24) - Foreign databases on the Internet (eg "Medline"), medical portals - ̤Medical representatives - ̤Congresses, conventions, stationary conferences, interactive conferences / on-line training - Information from the Ministry of Health, reports of the Agency for Health Technology Assessment and Tariffs - Clinical guidelines of medical associations  1. **Which of the factors has the greatest impact on your decision regarding the type of drug prescribed to the patient (please divide the pool of 100 points between the following answers, assuming that 100 has the greatest and 0 the least influence on the prescribed preparation):**  - Data related to the use of the drug (indications, side effects, interactions, etc.) - Guidelines that provide the best practices currently known - Cots of pharmacotherapy |
